# Cancer of the female lower genital tract

**DOI:** 10.1038/sj.bjc.6600357

**Published:** 2002-06-17

**Authors:** Allan MacLean

**Affiliations:** Royal Free Hospital, University College London, UK

## Abstract

*British Journal of Cancer* (2002) **86**, 1972. DOI: 10.1038/sj.bjc.6600357 www.bjcancer.com

© 2002 Cancer Research UK

## 

The statistics of ‘Cancer of the Female Lower Genital Tract’ are that there are 19 chapters, contained within 308 pages, from 28 contributors or co-authors to cover cancers of the cervix, vagina and vulva and certain rare tumours. The editors, Patricia J Eifel and Charles Levenback, are respectively Professor of Radiation Oncology and Associate Professor of Gynaecologic Oncology, University of Texas, MD Anderson Cancer Center at Houston, Texas, USA. Seven of their co-contributors are also based at the MD Anderson, and all the others (except Evanthia Kostopoulou of Thessaloniki, Greece) are North American. The book comes as one of a large series ‘Atlas of Clinical Oncology’ of the American Cancer Society and published by B.C. Decker, is accompanied by a CD ROM, and is retailed at £95.00 in the UK.

The book has considerable North American orientation – the demographics for vulval (vulvar) and vaginal cancer are for the USA, although a more global picture is included for the cervix. Some of the concepts or terminology are old – the term ‘dystrophy’ introduced by Professor Jeffcoate of Liverpool in the 1960s does little to facilitate an understanding of the aetiology of vulval cancer, and ‘smoking’ and ‘carcinogens from tobacco’ are barely mentioned under vulval and cervical cancers. The chapter on molecular biology gives a satisfactory review but its discussion of the role of angiogenic factors in the transition from pre-invasive to invasive lesions of the cervix and vulva is brief. It mentions the work of the Cardiff group on vaccine therapy for cervical (and vulval) neoplasia but misses opportunities to speculate how manipulation of immune response, cycle cell activity (by reversing HPV E6 proteins inhibitory effect on p53), potentiation of other tumour suppressor genes and anti-angiogenesis might provide therapy in the future.

I enjoyed the chapters on Anatomy and Diagnostic Imaging because of the quality of the figures/illustrations. The computerised tomography and magnetic resonance imaging, with appropriate labelling, have been selected to demonstrate relevant oncological challenges. The chapters on radiation therapy for invasive cervical, vaginal and vulval cancer are also well illustrated. Discussion includes concepts of technique, dose and dose rate, fractionation and target volume and is valuable for the gynaecological oncologist who is less familial with how radiation is prescribed. The chapter on surgery for vulval cancer includes comments on excision margins, alternatives to managing clitoral tumours, sentinel node identification and lymphatic mapping, and plastic surgical techniques.

The final chapter, appropriately, is on palliative care, and describes salvage therapy, as well as the principles of palliation, giving bad news and a table with ‘palliative pearls’ – the use of tables throughout the text provides helpful summaries.

Does the CD ROM disc add anything extra? I was disappointed because it provides a computerised version of the book; there are no computer-assisted learning exercises or interactions. Some may prefer scrolling through text on the screen but I would prefer reading the book.

I do not regard this as a compulsory purchase for trainees in gynaecological oncology but each training centre should buy a copy.

